# Cell-Mediated Immune Responses and Immunopathogenesis of Human Tick-Borne Encephalitis Virus-Infection

**DOI:** 10.3389/fimmu.2018.02174

**Published:** 2018-09-26

**Authors:** Kim Blom, Angelica Cuapio, J. Tyler Sandberg, Renata Varnaite, Jakob Michaëlsson, Niklas K. Björkström, Johan K. Sandberg, Jonas Klingström, Lars Lindquist, Sara Gredmark Russ, Hans-Gustaf Ljunggren

**Affiliations:** ^1^Department of Medicine Huddinge, Center for Infectious Medicine, Karolinska Institutet, Karolinska University Hospital, Stockholm, Sweden; ^2^Department of Infectious Diseases, Karolinska University Hospital, Stockholm, Sweden; ^3^Unit of Infectious Diseases, Department of Medicine Huddinge, Karolinska Institutet, Karolinska University Hospital, Stockholm, Sweden

**Keywords:** cell-mediated immunity, flavivirus, NK cells, T cells, tick-borne encephalitis, tick-borne encephalitis virus, viral immunopathogenesis

## Abstract

Tick-borne encephalitis virus (TBEV) is a flavivirus that belongs to the *Flaviviridae* family. TBEV is transmitted to humans primarily from infected ticks. The virus causes tick-borne encephalitis (TBE), an acute viral disease that affects the central nervous system (CNS). Infection can lead to acute neurological symptoms of significant severity due to meningitis or meningo(myelo)encephalitis. TBE can cause long-term suffering and has been recognized as an increasing public health problem. TBEV-affected areas currently include large parts of central and northern Europe as well as northern Asia. Infection with TBEV triggers a humoral as well as a cell-mediated immune response. In contrast to the well-characterized humoral antibody-mediated response, the cell-mediated immune responses elicited to natural TBEV-infection have been poorly characterized until recently. Here, we review recent progress in our understanding of the cell-mediated immune response to human TBEV-infection. A particular emphasis is devoted to studies of the response mediated by natural killer (NK) cells and CD8 T cells. The studies described include results revealing the temporal dynamics of the T cell- as well as NK cell-responses in relation to disease state and functional characterization of these cells. Additionally, we discuss specific immunopathological aspects of TBEV-infection in the CNS.

## Introduction

Tick-borne encephalitis virus (TBEV) is a flavivirus that belongs to the *Flaviviridae* family. Flaviviruses comprise many human pathogens including the commonly known Dengue virus (DENV), Japanese encephalitis virus (JEV), West Nile virus (WNV), Yellow fever virus (YFV), and Zika virus (ZIKV) (1). With respect to TBEV, three subtypes of the virus exist: European (TBEV-Eu), Siberian (TBEV-Sib), and Far Eastern (TBEV-FE) (2).

**Figure 1:**
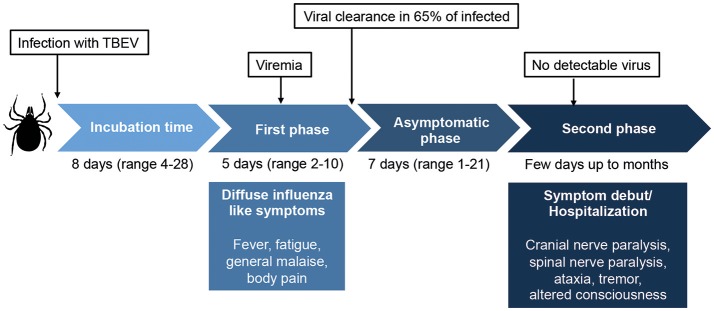
Overview of the classic biphasic disease-pattern of human TBEV infection. The viremic first phase includes influenza-like symptoms and occurs around 1 week after virus transmission. It is estimated that 65–70% of infected individuals clear the virus after this phase, but for one third of the patients, an asymptomatic disease phase follows before the second phase of disease begins. In this phase, symptoms of meningitis or encephalitis occur, including fever, headache, tremor, nystagmus, altered state of consciousness, cranial nerve paralysis, and spinal nerve paralysis. Classically, no virus is detected in sera or plasma in the second phase of disease. Around 30% of patients that enter the second phase of disease will suffer from long lasting sequeale, with a decreased quality of life. Figure compiled from Lindquist and Vapalahti (3), Taba et al. (4), and Haglund and Gunther (6).

TBEV is transmitted to humans primarily from infected ticks, mainly from the *Ixodes* family. The virus can also be transmitted from unpasteurized dairy products from infected livestock (3–5). Infection with TBEV causes tick-borne encephalitis (TBE), an acute viral infection that affects the central nervous system (CNS) with often severe long-term neurological consequences (3, 4, 6, 7). The first TBE-like disease was described as early as in the eighteenth century in Scandinavian church records (8). Traditionally, the disease is described as a syndrome with a biphasic course beginning with an influenza-like illness followed by a second neuroinvasive phase with neurological symptoms of variable severity, ranging from meningitis to severe meningoencephalitis with or without myelitis (3, 4, 6) (Figure 1). It shall be noted, however, that also monophasic patterns of disease development have been described (9). Upon infection, virus is detected in serum in the first phase of the disease but rarely in the second phase (10).

Due to increased geographic distribution of TBEV as well as a marked increase in morbidity in many areas, TBEV-infection has more recently caught attention as a public health problem. TBE is now observed in large parts of Europe as well as in northern Asia (3, 4). The main risk areas for TBE in Europe are primarily parts of central and eastern Europe as well as the Baltic and Nordic countries. With respect to central Europe, risk areas extend from Switzerland in the west into northern Italy and the Balkan countries (11). The incidence of TBEV-infection in endemic countries varies from year to year (12–14), however, an overall upsurge has been reported in certain parts of Europe, including the borders between Austria, Slovenia, and Italy (15, 16). These changes have been related to climatic, ecological, environmental, and socioeconomic factors that all can lead to an increased risk of human exposure to infected ticks (17–20).

The total number of annual cases has been estimated to be up to 13,000, and as such the infection constitutes the most important tick-borne viral disease (4). More than 30% of patients with clinical symptoms from TBEV-infection develop prolonged sequelae, some of which may become life-long including neuropsychiatric symptoms, severe headaches, and a general decrease in quality of life (3, 4, 6, 7). The mortality rates differ between the strains. Infection with the Far Eastern strain (TBEV-FE) has a mortality rate of 5–35%, whereas the other two strains (TBEV-Eu and TBEV-Sib) have mortality rates of 1–3% (3, 4). There is no specific treatment (e.g., antivirals) for TBE; rather, symptomatic treatment is the only available option (3, 4, 9).

Of importance, TBE may be prevented by vaccination. There are in total four licensed vaccines to TBE. Two vaccines based on TBE-Eu subtype are licensed in Europe and two are licensed in Russia. Additionally, a TBEV-vaccine based on the Far Eastern subtype is produced and marketed in China. All vaccines are based on formalin-inactivated strains of TBEV (3, 4, 21, 22). In areas where the disease is highly endemic, WHO recommends that vaccination should be offered to all groups above 1 year of age (4, 23). Primary vaccination against TBE includes three doses of the vaccine within the first year, followed by revaccinations every third to fifth year to maintain immunity. Vaccination is generally considered effective and TBE incidence has decreased substantially in TBEV-endemic regions with successful vaccination-programs (24). Randomized controlled trials in large populations have shown high immunogenicity with often-strong antibody production and acceptable rates of adverse events following vaccination (25–28). Breakthrough TBE after vaccination is generally considered rare (4). However, over the last years, vaccine failures have been reported, in particular in middle-aged and elderly individuals, who have completed the primary vaccination (29–31).

Infection with TBEV triggers humoral and cell-mediated immune responses. A confirmed diagnosis of TBE is established by the detection of specific IgM and IgG in serum. IgM antibodies have been observed in sera very early in symptomatic TBE disease, whereas IgG antibodies peak in the convalescent phase of disease (32). IgG antibodies can persist over lifetime and prevent TBE (4, 33). Early after clinical disease onset, TBEV-specific antibodies can also be found in the cerebrospinal fluid (CSF) (32, 33). In contrast to the humoral immune response, the cell-mediated immune responses elicited to natural infection have been rather poorly studied until recently. The latter responses may contribute both to host resistance against infection as well as to pathological reactions affecting the target organ of the virus, i.e., primarily the CNS.

Here, we review recent progress in studies of the cell-mediated immune response to human TBEV infection. A particular emphasis is devoted to natural killer (NK) cell- and T cell-mediated responses. Responses to TBEV are discussed in context of cell-mediated immune responses toward other flavivirus infections. We also discuss some immunopathological aspects of TBE with a particular emphasis on cell-mediated immune reactions in the CNS. Cell-mediated immune reactions in the CNS may contribute to neural damage with severe consequences of brain function, and could in the worst cases lead to fatal outcome. First, however, some aspects of the TBEV itself are covered.

## TBEV and other flaviviruses

All flaviviruses are enveloped and have a positive-sense single stranded RNA genome, which *per se* acts as messenger RNA upon entrance in the host cell. The RNA encodes for a polyprotein, which is co- and post-translationally cleaved by viral and cellular proteases into three structural proteins; capsid (C), precursor membrane (prM) and envelope glycoproteins (E), and seven non-structural proteins including NS1, NS2A, NS2B, NS3, NS4A, NS4B, and NS5 (34). Flaviviruses enter the cell through clathrin-dependent endocytosis upon attachment of the E protein to a receptor. Heparan sulfate has been identified as such receptor for TBEV (35); however, there are most likely also other yet not identified receptors for the virus. Following cell entry, the flavivirus is delivered to endosomes (36), in which the low pH triggers the E protein to fuse with the endosomal membrane and the nucleocapsid is released into the cytosol. The assembly of immature flavivirus virions (36–38), including TBEV (39, 40), occurs in the ER, and the viral particles are transported to the Golgi apparatus. The virion particles are immature until the envelope protein is rearranged and prM is cleaved by the host enzyme furin in the acidic environment in the Golgi apparatus. Immature particles are non-infectious and proteolytic cleavage of prM is a prerequisite for viral infectivity. However, studies have shown that complete cleavage of prM is not necessary for viral infectivity (41–43).

In general, species of flaviviruses have many similarities, but their preferred host cells differ. TBEV is shown to replicate 10,000-fold higher in human neuronal cells as compared with epithelial cells (44). A similar infection pattern has recently been shown for ZIKV (45).

## NK cells

NK cells are innate lymphocytes, though recent studies have revealed “adaptive” features of these cells (46, 47). They are perhaps best known for their ability to kill virus-infected and tumor cells. NK cell cytotoxicity is regulated by the expression of numerous activating and inhibitory receptors that sense ligands on neighboring healthy and altered cells. Several activating receptors recognize molecules that are up-regulated on cells during conditions of cellular stress, such as viral infection and transformation [reviewed in (48, 49)] whereas many inhibitory receptors, e.g., human killer cell Ig-like receptors (KIR) bind to HLA class I molecules. Additionally, NK cells have an important role in producing cytokines and chemokines, as well as by other means interacting with other immune and non-immune cells.

Human NK cells are classically defined as CD3^−^ (T cell receptor negative), CD56^+^ cells and represent about 15% of peripheral blood lymphocytes. These cells have for long been divided into two main subsets; CD56^bright^ and CD56^dim^ cells (50). The CD56^bright^ NK cells are thought to be less mature and are commonly known as primarily cytokine-producing cells with low cytotoxic ability, whereas CD56^dim^ NK cells are best known for their potent cytotoxic activity upon target cell recognition (51). However, the latter are also ample cytokine producers upon interaction with target cells (51). Both “natural” and antibody-mediated NK cell cytotoxicity is mediated by exocytosis of cytoplasmic granules containing perforin and granzymes (52). Cytotoxic responses may also to various degrees involve TRAIL- and Fas-ligand-mediated induction of apoptosis (53, 54). CD56^dim^ NK cells frequently express CD16 (FcγRIII), KIRs, and CD57, which regulate their function and define distinct stages of NK cell maturation (55), whereas CD56^bright^ NK cells largely lack expression of these molecules.

## The role of NK cells in human TBEV infection

Direct evidence for a protective role of NK cells has been found in experimental models of several viral infections, including cytomegalovirus and influenza, and a number of studies have indicated that they play a role also in protection against viral infections in humans. For example, NK cell-deficiencies in humans result in severe herpes virus infections in childhood and adolescence (56). NK cells may also have a protective role in human TBEV-infection. At the same time, responses mediated by these cells may be associated with development of symptoms in the course of TBEV-infection. Although there is only little known about NK cells in TBE, NK cells have been detected in CSF of patients with TBE (57), an observation that indicates transmigration through the blood brain barrier (BBB).

**Figure 2:**
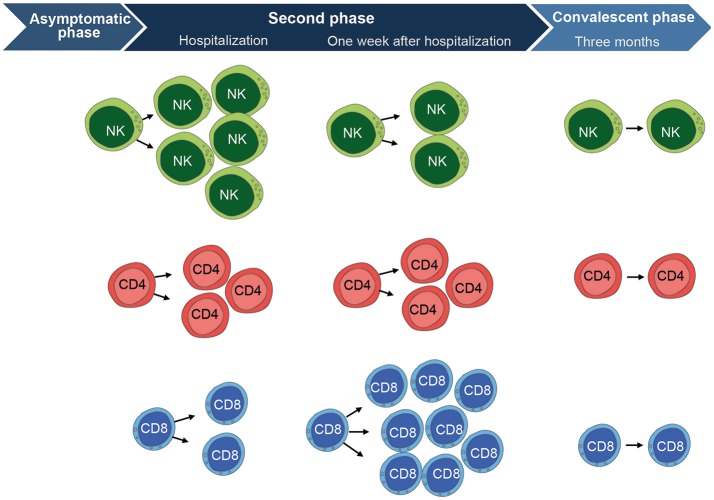
Overview of the cell-mediated immune response to TBEV-infection. CD56^dim^ NK cells (mainly highly differentiated CD57^+^ cells) are highly activated at the time of hospitalization during the second phase of disease. They express significantly higher levels of Ki67, CD38, and produce less cytokines in response to target cells as compared to the convalescent phase. NK cells then become fully normalized, comparable to healthy control levels, at the convalescent phase as assessed 3 months after hospitalization. CD4 T cells show a similar pattern as the CD56^dim^ NK cells. They are activated at hospitalization and express significantly higher levels of Ki67, CD38, HLA-DR, and Granzyme B as compared to the convalescent phase. CD4 T cells have retracted to normal healthy control levels at the convalescent phase. CD8 T cells show a different pattern and peak in activation at 1 week after hospitalization. They express significantly higher levels of Ki67, CD38, HLA-DR, Granzyme B, Perforin, PD-1, T-bet, and Eomesodermin as compared CD8 T cells at the convalescent phase. The CD8 T cell activation subsequently return to normal healthy control levels at the convalescent phase, just as is the case for CD4 T cells and NK cells. Figure compiled from Blom et al. (58) and Blom et al. (59).

To gain a better understanding of the NK cell response to human TBEV-infection, we recently performed a longitudinal study providing an in-depth analysis of the human NK cell response to acute TBEV-infection in a well-defined cohort of TBE patients. The study had an emphasis on NK cell responses during the second stage of disease from which clinical samples were available. NK cell activation, as measured by expression of the proliferation marker Ki67, was apparent at the time of hospitalization (58) (illustrated in Figure 2). Concomitant with the increase in NK cell activation in the acute stage of disease, augmented levels of IL-12, IL-15, IL-18, IFN-γ, and TNF were detected in patient plasma. In parallel with high levels of activation, the activated NK cells expressed less perforin, granzyme B, and Bcl-2. By 3 weeks after hospitalization, the NK cell activation decreased to levels seen in healthy controls. This TBEV-induced NK cell activation was restricted predominantly to more differentiated CD57^+^CD56^dim^ NK cells. Functionally, CD56^dim^ NK cells responded poorly to target cells at the time of hospitalization, but they recovered functional capacity to healthy control levels during the convalescent phase. The poor functionality of NK cell responses was exclusive for target cell recognition, since NK cell responses induced by IL-18 and IL-12 remained unchanged throughout the disease (58).

## NK cell responses toward other acute flavivirus infections in humans

To be able to interpret the above-mentioned NK cell responses to acute TBEV infection, it is important to understand NK cell responses to other acute virus infections, including acute flavivirus infections. In this respect, NK cells have to various extent been studied *ex vivo* in other acute flavivirus infections, including DENV (60, 61) and WNV (62, 63) as well as hepatitis C virus (HCV), a distant relative within the *Flaviviridae* family (64, 65). NK cells have also been studied after vaccination with the live attenuated YFV 17D vaccine (66–68). They have been suggested to influence disease severity and outcome, and to contribute to viral control in these infections, even though underlying mechanisms are not well studied.

In this context, it was observed that the absolute number of NK cells in patients with a mild form of infection with DENV was higher as compared to patients with the more severe form of the infection, dengue hemorrhagic fever (DHF) (69). Reduced numbers of NK cells in the circulation may be indicative of migration toward peripheral target organs. Furthermore, a higher frequency of NK cells expressing CD69 early on during the infection in children developing severe DHF has been reported (70). In recent studies of DENV-infection, we found NK cells to be robustly activated during the first week after symptom debut. Here, the response seemed to be confined largely to the CD56^bright^ subset of NK cells and less mature CD56^dim^ NK cells (our own unpublished studies). Noteworthy in the context of acute TBEV infection, activation of NK cells may also occur very early, even before the onset of symptoms. This possibility is supported by the observation that the highest levels of NK cell activation in most TBEV infected patients were observed already at the time of hospitalization (58). This notion is corroborated in studies of YFV vaccinated individuals. An early response by NK cells was observed in study subjects vaccinated with YFV-17D, where expression of both Ki67 and CD69 was increased on NK cells as early as 1 week after vaccination (66). Accumulation of adaptive-like NK cells expressing the activating receptor CD94/NKG2C has been reported in some human viral infections (71–73); however, no expansion of NKG2C^+^ NK cells in blood has been observed in TBEV-infection or any other flavivirus infection (58). It can, however, not be excluded that this type of expansion could occur locally, e.g., at the site of infection.

In addition to the observed activation of NK cells *in vivo* in different flavivirus infections, a protective role of NK cells is also supported by *in vitro* data. For example, primary activated human NK cells have been shown to inhibit WNV-infection of Vero cells (63) and IFNα-activated NK cells can kill HCV-infected hepatoma cells *in vitro* (65). In addition, flavivirus-infected target cells have been reported to display virus-mediated up-regulation of MHC class I (74), and could thereby theoretically evade lysis from NK cells by engaging inhibitory receptors. The dampened NK cell responses to target cells in acute TBEV-infection further support this notion (58). On the other hand, increased MHC class I expression could result in enhanced T cell responses. In such a scenario, one may speculate that flaviviruses may have been driven more toward escape from innate immunity rather than from adaptive T cell immunity (75).

## T cells

In contrast to NK cells, CD8 and CD4 T cells recognize specific foreign peptide sequences presented by HLA class I and II molecules, respectively (76). Like NK cells, major functions of CD8 T cells are to kill infected cells through the release of perforin and granzymes, and to secrete cytokines such as IFN-γ, TNF, and IL-2. The cytotoxic T cell response to acute infection can typically be divided into three phases; priming and expansion, resolution and contraction, and memory formation. During the first phase, naïve CD8 T cells divide and differentiate into effector cells acquiring high cytotoxic ability (77). Following viral clearance, the effector T cell population contracts and the majority of the pathogen-specific T cells enter apoptosis. A small pool of pathogen specific T cells (5–10%) survives as memory cells in the third stage (78). Memory T cells are a principal component of immunity against intracellular pathogens such as viruses. They are distinguished by their capacity to survive long-term, and undergo rapid and robust proliferation and acquisition of effector function upon antigen re-exposure (78). Memory T cells can vary in their phenotype, localization, and function allowing them to protect the host against a broad array of potential insults.

Distinct stages of CD8 T cell differentiation are defined by the expression of specific surface markers such as the isoforms of CD45 and expression of the homing receptor CCR7. These stages of differentiation are useful in the characterization of responses to, e.g., anti-viral responses. The set stages define CD45RA^+^CCR7^+^ as naive (T_N_), CD45RA^−^CCR7^+^ as central memory (T_CM_), CD45RA^−^CCR7^−^ as effector memory (T_EM_), and CD45RA^+^CCR7^−^ as effector memory RA (T_EMRA_) CD8 T cells (79, 80). T_CM_ cells primarily reside in secondary lymphoid organs, possess the greatest proliferative potential among the memory T cell subsets and can rapidly expand and differentiate following re-challenge. T_CM_ cells have higher sensitivity to antigenic stimulation, are less dependent on co-stimulation and provide better feedback to DCs and B cells compared to T_N_ cells. T_EM_ cells can migrate between tissues and secondary lymphoid organs and provide immune surveillance.

## The role of T cells in human TBEV infection

Due to difficulties in identifying the acute phase of viral infection in humans, T cell responses to viral infections have to a large extent been addressed in studies of pathogens causing chronic infections such as HIV-1, EBV, HCV, and CMV (81–85). Such responses can be very robust, as exemplified by the massive clonal expansion of antigen-specific CD8 T cells seen in many infections (83, 85). Based on these studies, it has also become clear that the resulting populations of human CD8 T cells display striking phenotypic differences, as determined by the expression profiles of surface markers (79–81). In contrast to many other infections, including some flavivirus infections (86–89), there are only few studies of T cell responses to TBEV-infection in humans. This hold true for acute as well as cases with prolonged TBE disease. Noteworthy, however, one report has shown that TBEV-specific CD4 T cells from naturally infected patients show a higher level of polyfunctionality in response to antigen in the convalescent phase of disease, as compared to TBE-vaccine specific T cells (90).

The general lack of studies more systematically characterizing the human T cell response to TBEV-infection prompted us to study the primary T cell-mediated immune response in patients diagnosed with TBE with a particular emphasis of CD8 T cells (59). Similar to our study on NK cells (58), the T cell study focused on responses during the second stage of disease from which clinical samples were available. During this phase, CD8 T cells were strongly activated, as detected by increased expression of Ki67, within 1 week of hospitalization (illustrated in Figure 2). A large part of these CD8 T cells expressed high levels of perforin and granzyme B, and low levels of the anti-apoptotic protein Bcl-2. In contrast to CD8 T cells, CD4 T cells showed only low or at most moderate levels of activation. The TBEV-antigen specific CD8 T cells had a T_EM_ PD-1^+^ phenotype throughout the course of disease. TBEV-specific CD8 T cells were predominantly Eomes^+^Ki67^+^T-bet^+^ in the acute stage of disease. This pattern was replaced by an Eomes^−^Ki67^−^T-bet^+^ profile in the convalescent phase of disease. TBEV-specific CD8 T cells were mainly monofunctional in the acute stage of disease, and tended to become more polyfunctional in the convalescent phase when clinical symptoms retracted (59).

## T cell responses toward other acute flavivirus infections in humans

To be able to better interpret the above-mentioned T cell responses to acute TBEV infection, we compared the present results with T cell responses to other acute virus infections, including infections by other flaviviruses. The live attenuated YFV-vaccine strain can replicate after vaccination leading to a detectable viral load similar to a mild infection. Thus, this vaccine can be utilized as a controlled model to study mild acute viral infection in humans. CD8 T cells become activated within 1–2 weeks after vaccination with the YFV vaccine (86, 91, 92). YFV antigen-specific CD8 T cells predominantly display a T_EM_ PD-1^+^ phenotype, which transition into a T_EMRA_ PD-1^−^ memory phenotype (86). With respect to DENV-infection, a high level of functionality of DENV-specific T cells is associated with a better disease outcome (93). Similarly, patients hospitalized with (severe) TBE show a low level of T cell functionality in the acute stage of disease (59), indicating the importance of high function among virus-specific T cells for beneficial disease outcome. CD8 T cells have been shown primarily to respond with IFN-γ to JEV in asymptomatic JEV-exposed donors (87).

Activation of CD4 T cells with an optimal magnitude, specificity and kinetics may be a requirement for viral clearance and protective immunity. In the immune response induced by the YFV vaccine, activation of CD4 T cells (peak at 10 days after vaccination) precedes that of CD8 T cells, and this may be of importance to elicit strong immunological memory (86). Furthermore, CD4 T cell release of IFN-γ may have an impact on disease outcome since CD4 T cells, and not CD8 T cells, were shown to dominate the IFN-γ response in recovered Japanese encephalitis (JE) patients. In addition, a high quality polyfunctional CD4 T cell response can be associated with better disease outcome in JE patients (87). In murine models, a perforin-dependent mechanism by the CD8 T cells has been shown to clear WNV from infected neurons, thereby suggesting an immunopathological role of T cells in mice (88). In this context, it is of interest to note that TBEV-specific T cells have a high content of both perforin and granzyme B (59), but whether the same perforin-dependent mechanism is causing immunopathogenesis in acute infection with TBEV remains to be investigated.

## Cross-reactivity within the family of flaviviruses

Immunological cross-reactivity between TBEV and other species within the flavivirus family may also contribute to disease (94, 95). Antibody-dependent enhancement (ADE) is a described phenomenon that can occur when non-neutralizing antibodies facilitate virus entry into host cells, leading to increased infectivity in the cells. ADE is commonly observed *in vitro* in cell culture-based models (96), but it is questioned as to which degree this phenomenon occurs *in vivo*. A recent study from non-human primates *in vivo*, did not observe increased ZIKV titers after prior infection with heterologous flaviviruses (97). Protective cross-reactivity of flaviviruses has been reported as well, opposing increased pathogenesis upon pre-exposure to other species of flaviviruses (98). TBEV has been suggested to cause both pathogenic and protective cross-reactivity. Polyclonal sera against members of the TBE serocomplex (including TBEV, Kyasanur Forest disease virus, Omsk hemorrhagic fever virus, and Langat virus) enhance viral replication of TBEV *in vitro* (96). However, it has recently been shown that antibodies generated from TBEV infection or from the TBEV vaccine can mediate cross neutralization against other, if not most, of the members of the TBE virus complex (99). Furthermore, sera from some individuals vaccinated against TBEV and JEV neutralized WNV, and the neutralization was enhanced by YFV vaccination in some recipients (95), altogether indicating that previous flavivirus exposure may sometimes provide a degree of protection to new flaviviruses.

Cell-mediated immunity and cross-reactivity caused by TBEV and other flaviviruses has been less well studied. Recently, a study demonstrated that JEV- and JE vaccine-specific T cells cross-react with DENV (87, 100). In line with this, it was also recently shown that vaccination with YFV vaccine could induce ZIKA-specific T cells, thereby suggesting cross-protection of flavivirus-specific T cells (101). The latter phenomena opened up a discussion as to the possibility of utilizing the YFV vaccine to protect against Zika virus infection (101). In the present context, it remains to be investigated whether YFV vaccination elicits protective cross-reactive immunity also toward TBEV.

## Immunopathological aspects of TBEV infection in the CNS

In contrast to the significant interest in emerging infections such as the recent Zika pandemic [reviewed in (45, 102, 103)], studies of the immune response toward TBEV-infection as such, and TBEV-induced immunopathology in particular, have been rather limited.

Immune and none-immune mechanisms have been proposed contribute to the crossing of TBEV over the BBB and invasion of the CNS [reviewed in (104)]. Cytokines may facilitate this process. Cytokines such as TNF-a and IL-6 have an impact on endothelial cell permeability that may induce a BBB disruption (105, 106), leading to crossover of the virus into the CNS. A distinct mechanism by which the TBEV could possibly cross the BBB is the *Trojan Horse* mechanism (107), by which TBEV-infected immune cells such as dendritic cells, neutrophils, monocytes, macrophages, and T cells would migrate into the parenchymal compartment causing infection of neurons or other cells in the brain and the spinal cord. Yet, an alternative route is invasion via the olfactory epithelium (108, 109).

After the landmark discovery of the lymphatic system present in the meninges that connects the CNS to the peripheral blood (110, 111), the classical concept of the CNS as an immune privileged site has been replaced by a view of an immune regulated site. Hence, under normal conditions a continuous transmigration of lymphocytes, monocytes, DCs and macrophages occurs. They may serve to detect any kind of infection or injury in the brain [reviewed in (112)]. Similar to other CNS infections, increased frequencies of T cells have been reported in CSF of TBE patients (57). Hence, activated T cells are crossing the BBB in TBE; however, the role of T cells at this site not well understood (113, 114). On one hand they could contribute toward clearing viral infection but on the other hand they may mediate immunopathology within the CNS. Corroborating the latter speculations are findings in which granzyme B^+^ CD8 T cell infiltrates have been linked to cell-death in infected human neuronal tissue (113) and, in parallel, mice with CD8 T cell deficiency have been shown to have prolonged survival upon infection with TBEV compared to mice with adoptively transferred CD8 T cells to immuno-competent mice (114). Furthermore, studies of post-mortem tissue of TBE patients have shown a predominance of macrophages/microglia and CD3^+^ T cells (both CD4^+^ and CD8^+^) in brain parenchyma (113). As seen in other flavivirus infections, macrophages and microglia also play a role in tissue destruction in human TBE.

In relation to NK cells and their possible role in causing immunopathogenesis, it is of interest to note that also these cells have been detected in the cerebrospinal fluid (CSF), albeit in low numbers, in patients with severe TBE meningitis or encephalitis (57). Activated NK cells may be protective, but they may also, like T cells, take part in immunopathological reactions as they are known to participate in direct killing of infected cells, indirect killing through cytokines or chemokines, or by the recruitment of inflammatory cells into the tissues (115, 116). Although recent results support a role for NK cells in clinical TBEV-infection, more studies are needed to provide a better understanding of the role NK cells play in pathogenic processes of human TBE infection.

Knowledge and experience gained in the field of the immunopathogenesis of other diseases affecting the CNS and its immunological compartments could be helpful in understanding TBE-specific diseases patterns. For example, in multiple sclerosis (MS), an inflammatory disease with pathology affecting the CNS (117), the concentration of the Sphingosine-1-Phosphate (S1P) in CSF is elevated and S1P-signaling is altered. In MS, binding of S1P to S1P1-receptors expressed on lymphocytes leads to invasion of autoreactive T cells into the CNS, the latter contributing to the hallmarks of the disease including demyelination and neurodegeneration (118). Interestingly, during the phase of acute infection in TBEV-infected patients, the levels of S1P in blood and CSF are highly elevated (119). This increase might promote a proinflammatory response. An increased production of extracellular S1P can be regulated by modulators of the S1P pathway, such as fingolimod, which is an immunomodulatory drug used in the treatment of MS (118). Therefore, therapeutic options used in other CNS diseases that share common immunopathogenic mechanisms with TBE could be used as models to aid in the development of new strategies for TBE treatment.

## Concluding remarks

In the present review, we have focused our attention to recent insights into the cell-mediated immune response to human TBEV infection, with an emphasis on studies of NK cell and CD8 T cell mediated responses. Until recently, the latter have been poorly studied. As yet, however, much more needs to be learnt with respect to these responses and research in this area should be encouraged. We have also addressed some aspects of TBEV CNS pathogenesis, a process still far from understood in detail. Clearly, however, cell-mediated immune responses likely play an important role in this process. As TBE continues to be an increasing global health problem and challenge, much more research is needed into this emerging disease. Several areas of research of the TBEV itself, and the clinical disease TBE merit further studies. Not the least, the specific organ pathogenesis caused by TBEV and the immune response, including infiltrating immune cells, needs more investigation. Furthermore, the possibility of antiviral treatment and other possible treatment modalities needs much more thorough investigation to prevent disease development and the often severe sequeale following infection of humans with TBEV.

## Author contributions

KB, AC, and H-GL wrote the manuscript. All other authors provided valuable contributions and insights into the manuscript.

### Conflict of interest statement

The authors declare that the research was conducted in the absence of any commercial or financial relationships that could be construed as a potential conflict of interest.
